# The effects of valence and arousal on time perception in individuals with social anxiety

**DOI:** 10.3389/fpsyg.2015.01208

**Published:** 2015-08-17

**Authors:** Jung-Yi Yoo, Jang-Han Lee

**Affiliations:** Clinical Neuro-pSychology Lab, Department of Psychology, Chung-Ang University, Seoul, South Korea

**Keywords:** time distortion, threatening face, social anxiety, arousal, valence

## Abstract

Time distortion in individuals with social anxiety has been defined as the seemingly slower passage of time in social situations and is related to both arousal and valence. Consequently, adaptive behavior is disrupted and interpersonal situations avoided. We explored the effects of valence and arousal on time distortion in individuals with social anxiety. Participants were assigned to two groups, High Anxiety (HA) and Low Anxiety (LA), presented with four types of facial expression stimuli (positive-high arousal, positive-low arousal, negative-high arousal, and negative-low arousal), and asked to estimate the duration of stimulus presentation. Results indicated that, relative to other stimuli, the HA and LA groups perceived longer presentation for high-arousal negative and low-arousal positive stimuli, respectively. These findings suggest that anxious individuals’ time distortion was more severe in situations that evoked high arousal and involved negative emotion.

## Introduction

Anxious individuals perceive time as passing slowly in threatening situations (Bar-Haim et al., 2010). Research has shown that anxiety that is evoked by specific situations affects the perception of time. For example, individuals with spider phobia have been found to misperceive the time spent observing a spider as longer relative to that perceived by those without spider phobia (Watt and Sharrock, 1984). Accordingly, anxiety-based symptoms are associated with the feeling that the passage of time is slower than usual. Given the increase in anxiety-based symptoms (e.g., palpitations, sweating, and blushing) in social situations in individuals with social anxiety, they also perceive time as passing slowly. Moreover, a distorted perception of time leads to excessive anxiety in social situations, thereby aggravating the anxious state (Bar-Haim et al., 2010).

The internal clock model (Triesman et al., 1990), which explains the process of time perception in the general population, has also been used to examine this process in individuals with social anxiety. According to the internal clock hypothesis, individuals have two internal clocks: a pacemaker and an accumulator. The pacemaker emits a series of pulses that form temporal units at a constant rate, and the accumulator counts the pulses produced by the pacemaker. Finally, the summation of pulses forms a subjective time estimation, which is stored in working memory and compared with previously timed durations stored in reference memory. Individuals possess neural mechanisms that allow the accurate estimation of time; however, when arousal is increased, the perceived duration of an event is longer than its actual duration. This distorted perception of time is related to the pacemaker’s acceleration of the speed at which pulses are emitted.

Numerous studies have reported an effect of arousal on time perception (Wearden and Ferrara, 1995; Wearden et al., 2006). The participants in these studies were presented with an oval stimulus for short (e.g., 400 ms) and long (e.g., 1,600 ms) standard durations. Neutral and emotional (threatening, angry, happy, sad, and neutral) faces were then displayed for different durations, and participants were asked to categorize the durations of the presentation of faces as more similar to either the short or long standard duration. The results showed that participants perceived emotional faces to have been presented for longer periods relative to neutral faces. The distorted perception of the duration of the presentation of angry faces was replicated in other studies, in which it was perceived to be longer relative to that of neutral faces (Droit-Volet et al., 2004; Effron et al., 2006). These findings were explained by the generation of a higher level of time distortion with highly arousing faces, which supports the internal clock model. However, findings from these studies have since been questioned due to difficulty in discriminating between the effects of arousal and valence on time perception (Angrilli et al., 1997).

Emotion is another factor that influences the perception of time. Recent studies have led to increased interest in using standardized and validated emotional materials to clarify the effects of arousal and valence on time perception (Coan and Allen, 2007). For example, emotional stimuli exert different effects on the judgment of time according to the arousing and emotional aspects of stimuli (Angrilli et al., 1997). The researchers manipulated valence and arousal and asked participants to judge whether the durations of stimuli presentation were shorter or longer relative to the standard duration learned in practice trials. The results showed that high-arousal negative stimuli were judged to have been presented for longer durations relative to high-arousal positive stimuli, but low-arousal negative stimuli were judged to have been presented for shorter durations relative to low-arousal positive stimuli. These results could be interpreted as demonstrating an interaction between the arousing and emotional aspects of the stimulus and suggests that the effect of arousal on time perception is modulated by the valence of the stimulus.

Individuals with social anxiety experience a high level of arousal in social situations due to fear of negative evaluation from others (Dienstbier, 1989; Hoehn-Saric and McLeod, 2000). Therefore, they perceive their experiences to last longer relative to those of non-anxious individuals in social situations, and the perception of time passing slowly could shape their subjective experiences of anxiety. In addition, they attempt to avoid allocating their attentional resources to negative information that feels threatening (Dalgleish and Watts, 1990). This could also cause a distorted perception of time, as experiences increase emotion-driven responses, and their attentional resources are allocated to the passage of time.

The aim of this study was to investigate the mechanism underlying the effects of both arousal and valence on time perception in individuals with social anxiety. In each trial, participants viewed faces with emotional expressions (high-arousal positive, low-arousal positive, high-arousal negative, low-arousal negative, and neutral) and were required to provide a verbal estimate of the duration of the presentation of stimuli. In the high-anxiety group, we expected the durations of the presentation of high-arousal negative stimuli to be perceived as longer relative those of low-arousal negative stimuli, with no differences in the judgment of presentation duration between low- and high-arousal positive stimuli. In contrast, in the low-anxiety group, we expected that the durations of the presentation of low-arousal negative stimuli would be perceived as shorter relative to those of low-arousal positive stimuli, with no differences in the judgment of presentation duration between low- and high-arousal negative stimuli.

## Materials and Methods

### Participants

Initially, 420 participants were recruited from a university in Seoul, Korea. Of these, 40 were selected based on their Social Interaction Anxiety Scale (SIAS; Mattick and Clarke, 1998) scores. SIAS scores for the top and bottom 10% were allocated to the high-(HA) and low-anxiety (LA) groups, respectively. There were 20 participants in both the HA (10 women; mean SIAS score = 57.80, SD = 4.20) and LA groups (10 women; mean SIAS score = 10.30, SD = 5.60). SIAS scores differed significantly between groups, *t*(38) = –42.79, *p* < 0.05.

### Stimuli and Apparatus

The fixation display consisted of a white cross (Arial Unicode MS, size 40). The picture stimuli included 62 pictures selected from the Korea University Facial Expression Collection (KUFEC; Lee et al., 2006), classified into five categories based on two dimensions, arousal and valence: (1) high-arousal positive, (2) low-arousal positive, (3) high-arousal negative, (4) low-arousal negative, and (5) neutral. Thirty-six undergraduates were asked to rate the degree of arousal (i.e., calm-excitement) and valence (i.e., unpleasant-pleasant) experienced after viewing each picture, to identify the best examples in each category. Arousal and valence were rated using 7-point scales ranging from 1 (*calm*) to 7 (*excited*) for arousal and 1 (*very unpleasant*) to 7 (*very pleasant*) for valence. A total of 15 picture stimuli (i.e., angry, happy, sad, neutral) were selected and grouped into five categories, each containing three pictures, according to the arousal and valence rating scores for the top 20% of each category. The means and standard deviations of the ratings for each picture are shown in Table 1. Neutral stimuli were used as fillers; therefore, the data that was recorded for them were excluded from the statistical analysis. Each stimulus appeared at a size of 13 × 12 cm at the center of a 17-inch computer monitor. Stimulus presentation and data collection were controlled via eye-tracking equipment (iView XTM Red-IV Eye Tracking System), but the resultant data were excluded from analysis, as the eye-tracking equipment was used only to confirm that participants had seen the stimuli.

**TABLE 1:** **Means (SD) of the ratings for each picture**.

	**High-arousal**		**Low-arousal**		**Neutral**
	**Positive**	**Negative**	**Positive**	**Negative**	
Arousal	5.28 (1.20)	5.49 (0.88)	3.69 (1.08)	3.60 (0.82)	4.11 (0.85)
Valence	5.14 (1.39)	1.88 (1.00)	4.35 (1.04)	2.84 (0.84)	3.77 (0.61)

### Procedure

Participants provided informed consent and were seated in a physiological measurement room. They were instructed to place their chins on a chin rest at a distance of 50 cm from the 17-inch computer monitor. In the first 5 trials, neutral picture stimuli were presented for 2,000 s, 4,000 s, or 6,000 s. Each trial began with the presentation of the fixation cross on the screen for 800 ms. Following the fixation display, one of the five neutral face pictures appeared for 2,000 s, 4,000 s, or 6,000 s; these pictures were not used in the test phase. Participants were instructed to pay attention to the pictures and estimate the duration of the presentation of each picture without using chronometric counting strategies. They were also advised that a question would be displayed on the screen. Participants were then asked to perform a verbal estimation task, in which a verbal estimate of the duration of the presentation of each picture was provided in time units (e.g., 6 min 30 s). The training phase was followed by the test phase, without a break. The 15 picture stimuli were presented for 2,000 s, 4,000 s, or 6, 000 ms and appeared randomly to prevent order effects according to arousal and valence. During this phase, participants continued to provide verbal estimates of the duration of the presentation of each picture, as described above.

The test phase consisted of 90 trials split into two blocks of 45. Each picture was presented once within each block. There were two repetitions of the three durations in each block, with each picture randomly assigned to one of these durations. The entire experiment lasted approximately 30 min. Following completion of the experiment, participants were asked to rate the arousal and valence of each stimulus using a 7-point rating scale. They were debriefed and provided with monetary compensation for their participation.

### Data Analysis

#### Time Estimation Outcome—Modified Time-corrected Scores

For each participant, modified time-corrected (T-corrected) scores were obtained during trials in which the participant had responded verbally to each type of stimuli. Modified T-corrected scores were derived from time scores and calculated to adjust for time distortion in the general population (Kim and Lee, 2012): modified time-corrected = (estimated—actual duration of picture stimulus presentation) + 0.5. Modified time-corrected scores provide information concerning the direction and degree of time distortion. A score of 0 indicates that the duration of stimulus presentation is estimated accurately, and a score of < 0 shows that the estimated duration of stimulus presentation is shorter than the actual duration of stimulus presentation. Conversely, a score of > 0 shows that the estimated duration of stimulus presentation is longer than the actual duration of stimulus presentation.

## Results

**FIGURE 1:**
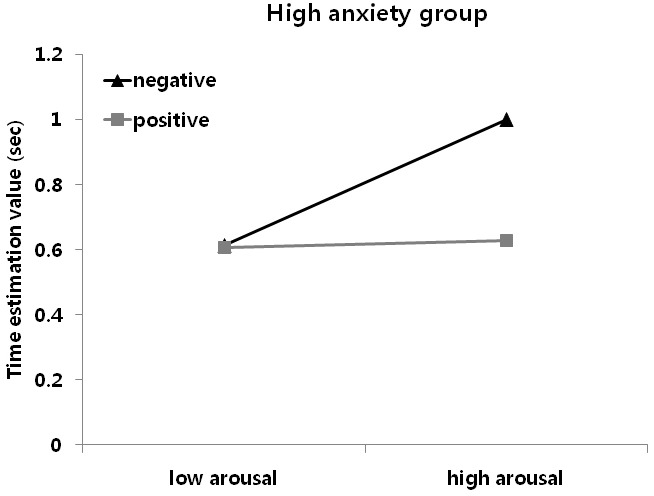
**Time estimation value (modified ***T***-Corrected) for each group**.

A 2 (Group: HA and LA) × 2 (Arousal: high and low) × 2 (Valence: positive and negative) mixed analysis of variance (ANOVA), with group as a between subject factor and arousal and valence as between-subject factors, was performed to identify differences in time perception between groups according to arousal and valence, using modified T-corrected scores (Figure 1). A significant effect of valence was observed, *F*(1, 38) = 26.37, *p* < 0.05, η^2^ = 0.41. Modified T-corrected scores were significantly higher for negative, relative to positive, pictures. A significant 2-way interaction between group and arousal was observed, *F*(1, 38) = 58.91, *p* < 0.05, η^2^ = 0.61, suggesting that patterns of modified T-corrected scores for arousal differed between the HA and LA groups. *Post hoc* tests showed higher rates of high, relative to low, arousal in the HA group, *t*(19) = –6.87, *p* < 0.001, and low, relative to high, arousal in the LA group, *t*(19) = 7.73, *p* < 0.001. A significant 3-way interaction between group, arousal, and valence was observed, *F*(1, 38) = 22.16, *p* < 0.05, η^2^ = 0.37, suggesting that patterns of time estimation differed according to arousal and valence between the HA and LA groups. Separate follow-up paired comparisons of arousal and valence were performed for each group to clarify the 3-way interaction. The HA group perceived the durations of the presentation of high-arousal negative pictures as significantly longer relative to those of high-arousal positive pictures, *t*(19) = –5.86, *p* < 0.05. In contrast, the LA group perceived the durations of the presentation of low-arousal positive pictures as significantly longer relative to those of low-arousal negative pictures, *t*(19) = –3.53, *p* < 0.05.

No significant effect of arousal, *F*(1, 38) = 0.80, *p* = 0.38, η^2^ = 0.02, or interactions between arousal and valence, *F*(1, 38) = 0.038, *p* = 0.85, η^2^ = 0.00, or group and valence, *F*(1, 38) = 1.96, *p* = 0.17, η^2^ = 0.05 were observed.

## Discussion

The results of the verbal estimation task were partly consistent with those reported by Bar-Haim et al., who used a time reproduction task (Bar-Haim et al., 2010) and found that, with a duration of 2 s, anxious subjects judged intervals of 2 and 4 s as longer when faces displayed a fearful expression relative to those for faces displaying a calm expression, while non-anxious individuals did not. There were no anxiety-related differences in time perception with stimulus exposure of 8 s. Therefore, regardless of the duration of the stimulus presentation, the HA group judged the durations of the presentation of angry faces as longer relative to those of neutral faces, but the LA group judged the durations of the presentation of almost all types of stimuli as shorter relative to those of neutral stimuli. In summary, the HA group overestimated time, while the LA group underestimated time. Specifically, the HA group was affected by valence for high-arousal stimuli, and the LA group was affected by valence for low-arousal stimuli.

Our results are consistent with the attentional-gate model (Zakay and Block, 1997) in a number of ways. The attentional-gate model proposes that (a) distorted time perception may occur according to whether attention is focused on time or the event; (b) when people focus on the event, it is difficult to perform an accurate internal calculation of time because of limited attentional resources; (c) when people focus on the event, they miss out on counting pulses, and the probability of time distortion, or time passing too quickly, increase; (d) conversely, when people focus on time, they may calculate internal time accurately, but this can increase the number of pulses counted due to other factors (e.g., high arousal or negative valence); and (e) this may trigger time distortion. Accordingly, individuals with social anxiety focused their attention on time and perceived it to be passing more slowly with increases in the number of pulses due to high arousal in social situations. However, further research is necessary to confirm whether they watch stimuli less than non-anxious individuals via eye-tracking equipment.

The present study aimed to examine differences in time perception between the LA and HA groups. Results showed that the LA group underestimated time, but the HA group overestimated time. This result supported the internal-clock model of time perception, which assumes that the accelerated pacemaker emits more pulses and causes time to be perceived as longer because of a higher level of arousal. However, the effect of arousal differed according to levels of anxiety. The LA group was affected by valence for low-arousal stimuli, but the HA group was affected by valence for high-arousal stimuli.

One interpretation of these results is that the duration lengthening effect of valence varied according to levels of arousal. Accordingly, when the LA group was exposed to both valence and arousal, they were affected by valence and subsequently overestimated time. Conversely, when the HA group was exposed to both valence and arousal, they experienced the additive lengthening effect of valence, because levels of arousal increased according to valence.

Previous studies examining time perception in anxious individuals have shown that this group overestimated time when they were exposed to threatening or angry pictures. Although these pictures could have influenced levels of arousal, facial expression pictures are a more direct method of influencing both valence and arousal. The present study used pictures of facial expressions to produce higher levels of arousal and valence. In addition, the study extends the findings of Bar-Haim et al. (2010) by replicating their findings concerning time perception in participants with high and low trait anxiety. By including the HA group of participants with social anxiety, we were able to explore differences in time perception between individuals with high and low social anxiety. When restricting our HA sample to socially anxious participants, differences in estimated time were observed between groups, suggesting that individuals with high social anxiety exhibit distorted time perception in social situations. However, further research is necessary to confirm this finding using a time estimation task to examine patients diagnosed with social anxiety disorder.

Interestingly, unlike previous studies, our results showed that all durations of stimuli presentation showed the same pattern of time perception. According to the vigilance-avoidance hypothesis, anxious individuals automatically detect threatening stimuli more rapidly, relative to neutral stimuli, with short presentation durations but exhibit greater avoidance of reactions to threat over time. Results of previous studies (Zakay and Block, 1997; Wilson and Macleod, 2003) were consistent with this hypothesis.

To summarize, this study aimed to examine differences between LA and HA groups in a valence- and arousal-inducing task. Our results suggest that the overestimation of durations of the presentation of facial expressions could be influenced differently according to levels of arousal in both anxious and non-anxious individuals.

### Conflict of Interest Statement

The authors declare that the research was conducted in the absence of any commercial or financial relationships that could be construed as a potential conflict of interest.
